# Transient Optic Disc Swelling After Laser Iridotomy for the Treatment of Acute Angle Closure Glaucoma

**DOI:** 10.7759/cureus.55765

**Published:** 2024-03-08

**Authors:** Shoma Tsuchiya, Shinji Makino

**Affiliations:** 1 Ophthalmology, Jichi Medical University, Shimotsuke, JPN

**Keywords:** optical coherence tomography, intraocular pressure, laser iridotomy, acute angle closure glaucoma, optic disc swelling

## Abstract

Acute angle closure glaucoma (AACG) is characterized by narrowing or closure of the anterior chamber angle of the eye. AACG typically presents in older, hyperopic patients who complain of blurred vision, ocular pain, halos around lights, headache, nausea, and vomiting. Optic disc swelling is known to be associated with intracranial hypertension, optic neuritis, anterior ischemic optic neuropathy, retinal vascular occlusion, and toxic optic neuropathy. There have been few reports of temporal relationships between laser iridotomy and optic disc swelling in patients with AACG.

In this case report, we present a case of AACG where optic disc swelling was developed after sudden lowering of the intraocular pressure (IOP) by laser iridotomy. A 65-year-old woman presented with left eye pain and poor vision for one day. Slit-lamp examination revealed conjunctival injection, corneal edema, and a nonreactive and mid-dilated pupil in the left eye. Her best corrected visual acuity (BCVA) was 20/20 in the right eye and counting fingers in the left eye. IOP was 10 mmHg in the right eye and 54 mmHg in the left eye. A diagnosis of left AACG was made. A peripheral laser iridotomy was performed. The details of the optic disc were difficult to observe due to corneal edema, but there were no obvious abnormalities. The next day, the BCVA was 20/60 and the IOP had decreased to 9 mmHg in the left eye. Fundus examination demonstrated optic disc swelling in the left eye. Spectral-domain optical coherence tomography (SD-OCT) scanning revealed optic disc swelling in the left eye. One week after treatment, the BCVA was 20/50 and the IOP was 10 mmHg in the left eye. Fundus examination and SD-OCT scanning revealed mild improvement of optic disc swelling in the left eye. Four weeks after treatment, the BCVA was 20/50 and the IOP was 10 mmHg in the left eye. Fundus examination and SD-OCT scanning revealed an improvement in optic disc swelling in the left eye. After performing laser iridotomy, it is necessary to pay attention to changes in the optic disc as well as the IOP.

## Introduction

Acute angle closure glaucoma (AACG) is characterized by narrowing or closure of the anterior chamber angle of the eye. AACG typically presents in older, hyperopic patients who complain of blurred vision, ocular pain, halos around lights, headache, nausea, and vomiting.

The visual outcome of patients with AACG depends on how early it is detected and treated. In the case of AACG, glaucomatous damage to the optic nerve is generally not reversible and can occur within hours, so it is important that the physicians urgently evaluate the patient and provide prompt diagnosis and treatment. AACG presents with abnormally elevated intraocular pressure (IOP). If the IOP does not decrease despite maximal medical therapy, laser iridotomy or lens extraction is performed.

Optic disc swelling is known to be associated with intracranial hypertension, optic neuritis, anterior ischemic optic neuropathy, retinal vascular occlusion, and toxic optic neuropathy. There have been previous reports of optic disc swelling associated with AACG [1-7]. However, reports of temporal relationships between laser iridotomy and optic disc swelling in patients with AACG are extremely rare [6,7]. Here, we report a case with AACG where optic disc swelling was documented after lowering the IOP by laser iridotomy.

## Case presentation

**Figure 1:**
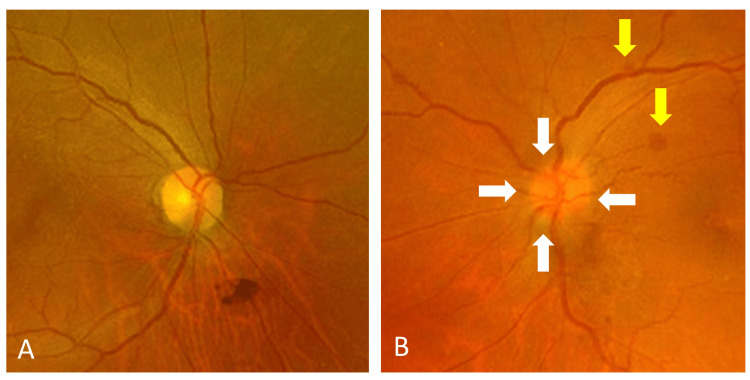
Photographs of the fundus of the right (A) and left (B) eyes one day after laser iridotomy. Note: Optic disc swelling (white arrows) and small retinal hemorrhages (yellow arrows) in the left eye.

A 65-year-old Japanese woman presented with left eye pain and poor vision for one day. Her personal and family histories, as well as the results of her physical examination, were unremarkable. Slit-lamp examination revealed left eye conjunctival injection, corneal edema, and a nonreactive and dilated pupil. The pupil diameter was 2.5 mm and 5.5 mm in the right and left eyes, respectively. Her best corrected visual acuity (BCVA) was 20/20 in the right eye and counting fingers in the left eye. IOP was 10 mmHg in the right eye and 54 mmHg in the left eye. The axial length was 22.81 mm and 22.55 mm in the right and left eyes, respectively. A diagnosis of left AACG was made. She was treated with intravenous acetazolamide and topical 4% pilocarpine. Cornea clarity was slightly improved, and a peripheral laser iridotomy was performed. The details of the optic disc were difficult to observe due to corneal edema, but there were no obvious abnormalities. The next day, the BCVA was 20/60 and the IOP had decreased to 9 mmHg in the left eye. Fundus examination demonstrated optic disc swelling and small retinal hemorrhage with venous tortuosity in the left eye (Figure 1B), which was not present in the fellow eye (Figure 1A).

**Figure 2:**
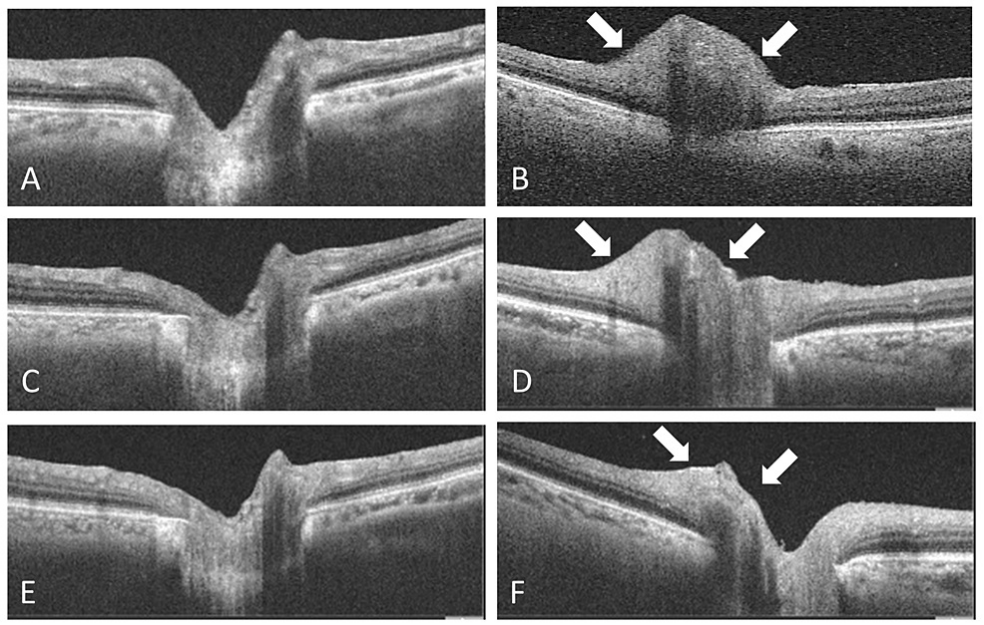
Spectral-domain optical coherence tomography of the right (A), (C), (E) and left (B), (D), (F) eyes. (A) and (B) One day after laser iridotomy, optic disc swelling persisted in the left eye (white arrows). (C) and (D) One week after laser iridotomy, optic disc swelling persisted in the left eye (white arrows). (E) and (F) Four weeks after laser iridotomy, optic disc swelling improved almost back to normal in the left eye (white arrows).

Spectral-domain optical coherence tomography (SD-OCT) scanning revealed optic disc swelling in the left eye (Figure 2B).

One week after treatment, the BCVA was 20/50 and the IOP was 10 mmHg in the left eye. Fundus examination and SD-OCT scanning revealed mild improvement of optic disc swelling in the left eye (Figure 2D). Goldmann visual field testing revealed mild visual field constriction in the left eye. Fluorescein angiography (FA) demonstrated no filling delay in the early phase, and hyperfluorescence of the optic disc was not observed in the late phase. Magnetic resonance imaging was otherwise normal.

Four weeks after treatment, the BCVA was 20/50 and the IOP was 10 mmHg in the left eye. Fundus examination and SD-OCT scanning revealed an improvement in optic disc swelling in the left eye (Figure 2F).

## Discussion

Here, we report a case of AACG where optic disc swelling was developed after the sudden lowering of the IOP by laser iridotomy. There have been previous reports of optic disc swelling associated with AACG [1-7]. However, reports of temporal relationships between laser iridotomy and optic disc swelling in patients with AACG are extremely rare [6,7]. Chai et al. [6] described the case of a 64-year-old man with optic disc swelling after laser iridotomy. According to their report, optic disc swelling was noted after laser iridotomy, which gradually resolved over the next five weeks. Kim et al. [7] described optic disc swelling in two patients with AACG. According to their report, optic disc swelling developed after laser iridotomy, which gradually resolved four months thereafter.

Optic disc swelling following AACG has been described in association with retinal vascular occlusion [1] and anterior ischemic optic neuropathy [2-5]. These diseases result in optic atrophy and irreversible vision loss in the affected eye. In our case, retinal vascular occlusion and anterior ischemic optic neuropathy were ruled out based on fundus and FA findings. Furthermore, our case demonstrates that transient optic disc swelling after AACG can have a relatively good visual outcome, and the optic disc swelling improved almost back to normal four weeks after laser iridotomy.

The suggested mechanism of optic disc swelling associated with AACG is compression of the vessels in the optic disc due to elevated IOP and the resulting ischemia in the optic nerve head [1, 8]. However, during elevated IOP, corneal edema usually interferes with accurate evaluation of the optic disc. In the present case, optic disc swelling was not observed during IOP elevation but was revealed after IOP lowering. We speculate that the optic disc swelling was associated with the sudden decrease in the IOP, leading to effusion from the choroidal capillaries in the prelaminar region and its collection in the loose glial tissue [6-8]. Alternatively, the optic disc may have swollen due to an overflow of axoplasmic flow after a sudden drop in the IOP [9].

## Conclusions

In this case report, we discussed one of the rare conditions after the sudden lowering of the IOP by laser iridotomy in a patient with AACG. After performing laser iridotomy, it is necessary to pay attention to changes in the optic disc as well as the IOP. Clinicians should be aware that when optic disc swelling is seen after the onset of AACG, choroidal effusion or axoplasmic overflow due to rapid lowering of the IOP is suspected, rather than other coexisting optic neuropathy.
